# Arthroscopic Saucerization of a Medial Discoid Meniscus in a Young Female Athlete: A Case Report

**DOI:** 10.7759/cureus.113879

**Published:** 2026-08-03

**Authors:** Travis Hodge, Brett Norris, Ryan Sorenson, Peter Schultz, Michael Dempewolf

**Affiliations:** 1 College of Osteopathic Medicine, Kansas City University, Kansas City, USA; 2 Department of Pathology and Anatomical Sciences, Kansas City University, Kansas City, USA; 3 Department of Orthopedic Surgery, Sano Orthopedics, Kansas City, USA

**Keywords:** discoid meniscus saucerization and repair, medial discoid meniscus, meniscus injuries, pediatric orthopedics, pediatric sports injury

## Abstract

A discoid meniscus is a congenital abnormality of the meniscus characterized by a disk-shaped configuration. Although medial involvement is uncommon, symptomatic cases may present in young, active individuals with mechanical knee symptoms that limit sports participation. A 15-year-old female athlete presented with chronic medial knee pain, mechanical symptoms, and an inability to fully extend the knee despite failed conservative management. MRI demonstrated a complete discoid medial meniscus with a central tear. Arthroscopy confirmed a discoid medial meniscus without significant peripheral instability. Arthroscopic saucerization was performed while preserving a stable peripheral rim. By the conclusion of the three-month follow-up, the patient had achieved improved pain control, full range of motion, and returned to activity without recurrent symptoms. Medial discoid meniscus is a rare cause of mechanical knee symptoms. Arthroscopic saucerization with meniscal preservation can result in favorable functional outcomes in young athletes.

## Introduction

A discoid meniscus is a congenital anatomic variant characterized by abnormal meniscal morphology, typically presenting as a thickened, disk-shaped structure rather than the normal semilunar configuration [1,2]. Discoid morphology most commonly involves the lateral meniscus, with reported prevalence estimates varying widely among populations and diagnostic modalities [3]. In contrast, a discoid medial meniscus is exceedingly rare, with a reported prevalence generally of less than 0.3% [4].

Patients with a discoid meniscus may remain asymptomatic; however, symptoms often develop in childhood or adolescence, particularly among athletes. Common presenting symptoms include activity-related knee pain, recurrent knee effusions, and mechanical symptoms such as catching or locking. In athletes, repetitive loading, pivoting, and cutting maneuvers may exacerbate symptoms or precipitate meniscal tearing or instability [4-7].

The embryologic etiology of discoid meniscus remains incompletely understood. Early theories proposed a failure of normal central meniscal resorption during fetal development. However, histologic and developmental studies suggest that abnormal morphogenesis and altered peripheral attachments may play a more important role [1,2]. Compared with the lateral meniscus, the medial meniscus demonstrates firmer capsular attachments and reduced mobility. These anatomic differences have been proposed as potential explanations for the markedly lower prevalence of discoid morphology in the medial compartment [1,3].

MRI remains the diagnostic modality of choice. Diagnostic criteria for a discoid meniscus include increased meniscal width with continuity between the anterior and posterior horns across multiple consecutive sagittal images, increased thickness, or abnormal meniscal contour [5,8]. Given the rarity of a discoid medial meniscus, diagnosis may be delayed or overlooked, particularly when symptoms are attributed to more common causes of medial compartment pathology.

Historically, total meniscectomy was performed for symptomatic discoid meniscus; however, long-term outcomes demonstrated an increased risk of early degenerative changes [9,10]. As a result, contemporary treatment strategies emphasize meniscal preservation whenever possible. Arthroscopic saucerization, consisting of partial meniscectomy to restore a near-normal contour while preserving a stable peripheral rim, has become the preferred surgical approach [3,11]. Recent studies have demonstrated favorable intermediate-term outcomes following saucerization and meniscal repair, further supporting contemporary meniscal-preserving treatment strategies [11]. When instability or peripheral tearing is present, saucerization may be combined with meniscal repair [6].

Despite an expanding body of literature regarding the discoid lateral meniscus, reports of the discoid medial meniscus remain limited and are largely confined to isolated case reports and small case series. Consequently, optimal management strategies are largely extrapolated from data on the discoid lateral meniscus. This case report describes a young female athlete with a symptomatic discoid medial meniscus treated successfully with arthroscopic saucerization. To our knowledge, this is among the few reported cases to provide a detailed sports medicine account of a symptomatic discoid medial meniscus in an adolescent female athlete. Although pediatric and adolescent female cases have been described previously, reports identifying the patient’s sport and documenting failed nonoperative management, meniscus-preserving saucerization, rehabilitation progression, and return-to-sport outcomes remain scarce. The purpose of this report is to highlight the clinical presentation, imaging findings, and surgical management of this rare condition while emphasizing the importance of meniscal preservation in the athletic population. Reporting individual cases contributes valuable insight into diagnostic considerations, surgical decision-making, and outcomes in this uncommon pathology [4,12].

## Case presentation

A 15-year-old female high school volleyball player presented with a 2.5-year history of right medial knee pain without a clear inciting injury. Although she continued to participate in competitive volleyball, she experienced pain with ambulation, an inability to achieve full terminal knee extension, and mechanical symptoms that progressively limited daily activities. Multiple courses of physical therapy resulted in transient symptomatic improvement; however, symptoms repeatedly recurred following completion of treatment and gradually worsened over time.

**Figure 1:**
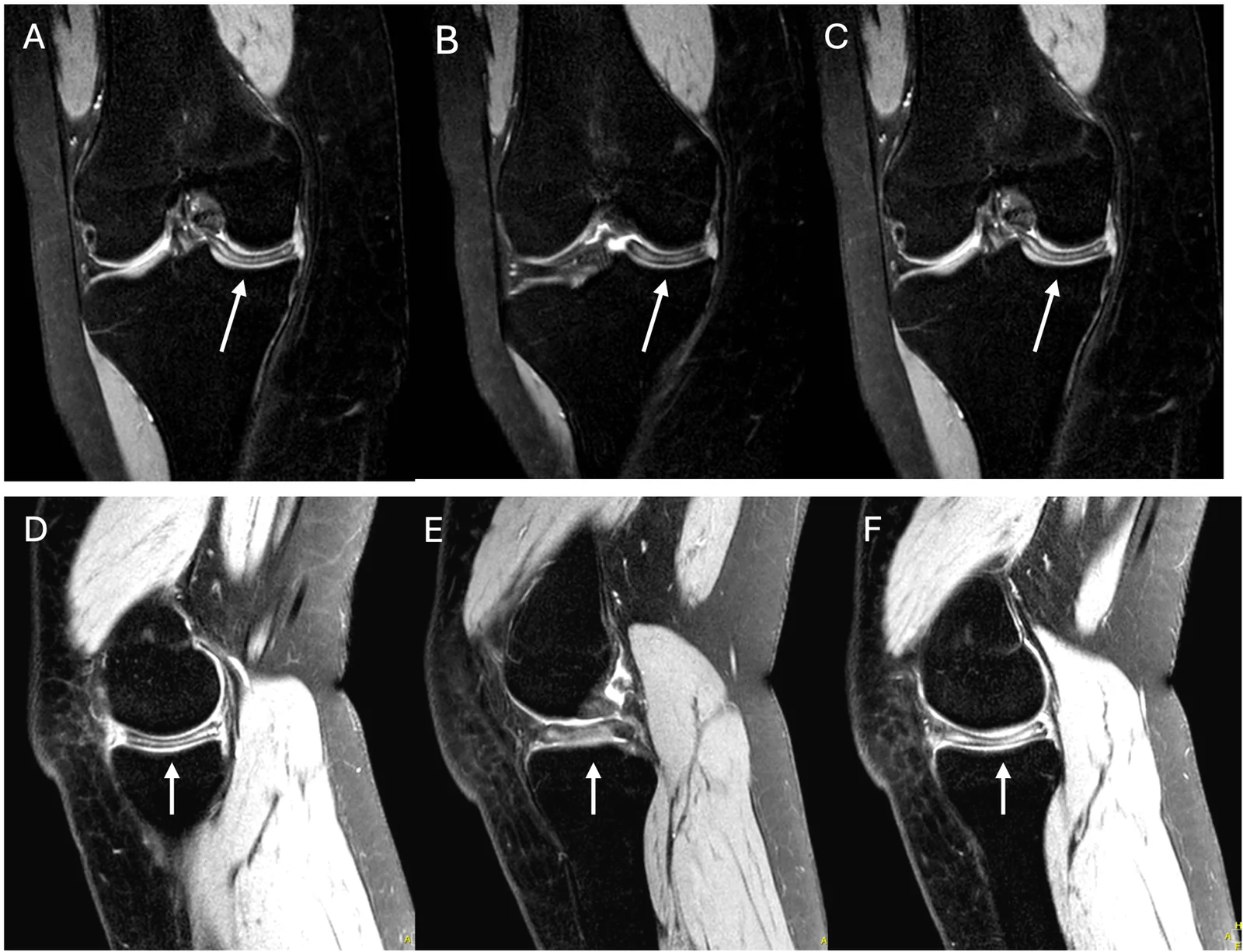
Preoperative MRI demonstrating a discoid medial meniscus. (A-C) Right knee coronal fluid-sensitive MRI sequences progressing from anterior (A) to posterior (C), confirming a complete discoid medial meniscus. A consistent slab-like width exceeding 5 mm forms a complete, continuous tissue bridge extending from the peripheral capsule to the intercondylar notch, thereby covering >80% of the medial tibial plateau (white arrows). This morphology persists without tapering across all three consecutive slices, satisfying the multislice diagnostic criteria. Linear high-signal-intensity fluid tracks transversely through the central body of the discoid meniscus. The horizontal tear originates near the central free edge of the discoid “slab” (C) and propagates peripherally along the horizontal midplane, completely dividing the body of the meniscus into superior and inferior laminae. The lateral meniscus demonstrates normal triangular morphology (white asterisk, A). (D-F) Right knee sagittal fluid-sensitive MRI sequences progressing from lateral (D) to medial (F). These images demonstrate classic features of a complete discoid medial meniscus (white arrows). The sequence reveals abnormal, continuous meniscal tissue spanning three consecutive slices, satisfying the sagittal diagnostic criterion of a continuous “bow tie” sign across three slices. Instead of transitioning normally from the peripheral body into distinct anterior and posterior triangular horns, the meniscal tissue remains thickened, block-like, and continuous throughout the joint compartment, confirming complete discoid morphology.

On physical examination, there was medial joint line tenderness and pain with terminal extension. Ligamentous examination revealed intact ligaments without evidence of instability. She lacked 10 degrees of terminal extension, and no clunk was noted with meniscal provocative testing. Medial clicking and pain were elicited with the McMurray and Thessaly tests. Given the persistent symptoms and failed conservative management, MRI was obtained and demonstrated a discoid medial meniscus with diffuse horizontal tearing of the discoid moiety and no displaced meniscal fragment, as shown in Figure 1. The lateral meniscus demonstrated normal signal intensity and morphology. The cruciate ligaments, collateral ligaments, and extensor mechanism were intact. The chondral surfaces were intact in all three compartments without focal or diffuse chondral loss. There was a physiologic amount of joint fluid and no popliteal cyst or popliteal fossa mass.

**Figure 2:**
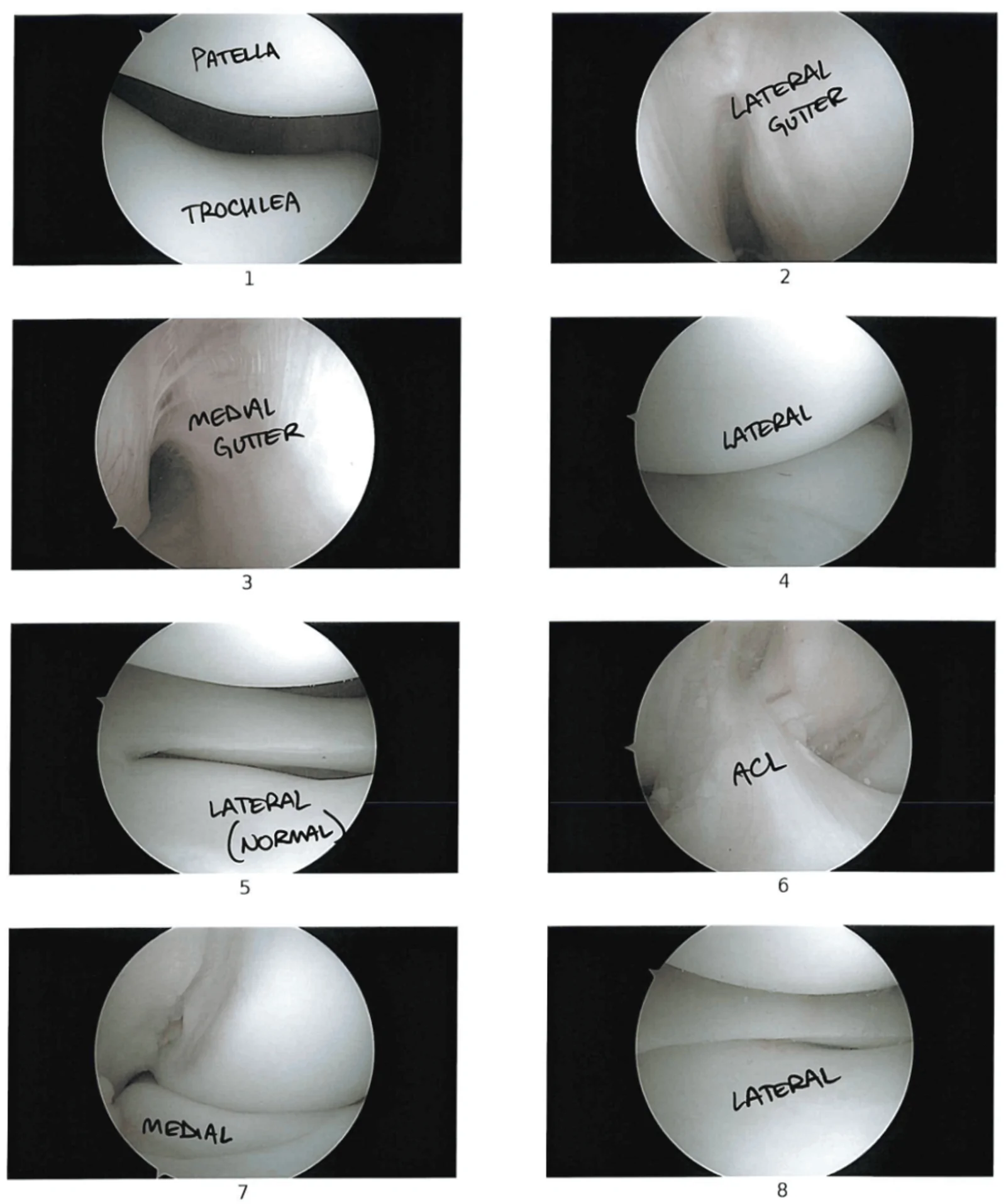
Arthroscopic view of the discoid medial meniscus occupying a greater-than-normal portion of the medial tibial plateau. ACL, anterior cruciate ligament

**Figure 3:**
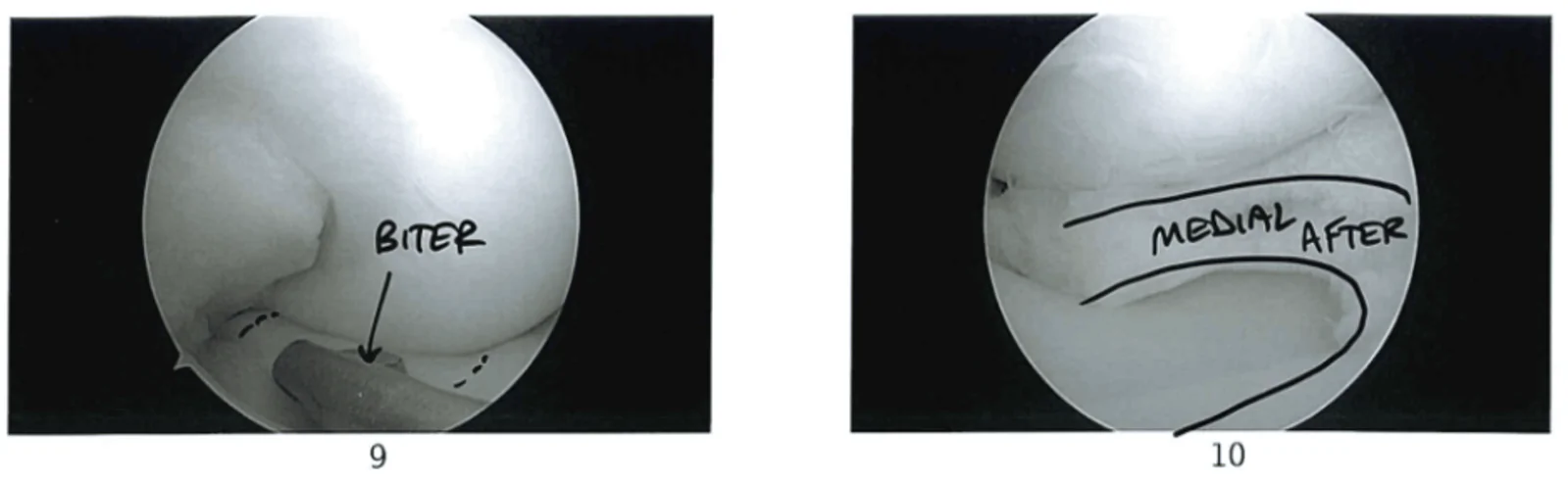
Arthroscopic images following saucerization demonstrating restoration of a more normal meniscal contour with preservation of the peripheral rim.

In response to the MRI findings, the patient underwent arthroscopic evaluation. Standard anterolateral and anteromedial arthroscopic portals were established. Diagnostic arthroscopy demonstrated a thickened discoid medial meniscus occupying a greater-than-normal portion of the medial tibial plateau, as shown in Figure 2. These findings, in conjunction with the preoperative MRI findings, confirmed the diagnosis of a complete discoid medial meniscus. Arthroscopic saucerization was then performed using biters and shavers to resect central tissue and restore a semilunar contour while preserving a stable peripheral rim of approximately 6-8 mm, consistent with previously described arthroscopic techniques [13], as shown in Figure 3. Following saucerization, the meniscal roots and remaining meniscal body were directly probed to assess stability and evaluate for residual tearing. No root avulsion, instability, or residual tear was identified, and repair was not indicated. The procedure was completed without complication.

Postoperatively, physical therapy was initiated twice weekly for 12 weeks, with a program emphasizing active and passive range of motion, progressive quadriceps and hip strengthening, and functional training for incline and decline activities. The patient was counseled to expect approximately six to eight weeks before being ready to return to athletic activity. Follow-up at six weeks demonstrated mild right quadriceps atrophy, full range of motion, and 5/5 strength on manual muscle testing. Despite normal manual strength, functional weakness contributed to difficulty with incline and decline activities, particularly stair descent; however, the patient denied pain with flat-ground ambulation. Gait was normal on flat ground without an antalgic pattern. At 12 weeks, further follow-up was deemed unnecessary because she no longer had difficulty with stairs, reporting full functional recovery and return to all activities of daily living without limitation. The patient was subsequently cleared by her physical therapist for a gradual return to sport as tolerated at 12 weeks, having regained functional recovery consistent with her preinjury status. Validated return-to-sport criteria (e.g., limb symmetry index and hop testing) were not available to report. As of eight months postoperatively, no complications or concerns had been reported to the clinic.

## Discussion

A discoid medial meniscus is a rare congenital variant of the knee that may present with a delayed diagnosis, particularly in young athletes with nonspecific symptoms such as knee pain, mechanical complaints, or intermittent effusions [3,4]. The present case shares several features with previously reported pediatric and adolescent cases of discoid medial meniscus, including medial joint line pain, mechanical symptoms, loss of terminal extension, and a prolonged symptomatic course. Kalenderer et al. described an 11-year-old boy presenting with knee pain and limited extension [14], whereas Iorio et al. reported a 13-year-old competitive football player with recurrent pain, effusions, popping, and locking during running and cutting activities [15]. In the latter case, an incomplete discoid medial meniscus was associated with tearing and peripheral instability and was therefore treated with saucerization and meniscal repair, with resolution of symptoms at the seven-month follow-up. A particularly comparable report described a 15-year-old male soccer player with three years of activity-related knee pain, locking, and popping. Arthroscopy demonstrated an extensive horizontal cleavage tear of the posterior horn that was considered irreparable, and saucerization with partial meniscectomy was performed while preserving the peripheral attachments [16].

In contrast, the current patient was a 15-year-old female high school volleyball athlete with a complete discoid medial meniscus and no peripheral instability on arthroscopic probing. Although central tissue was resected to restore a semilunar contour, the stable residual rim did not require repair. This distinction is clinically important because contemporary pediatric series support preservation of meniscal tissue through saucerization, with repair reserved for patients demonstrating peripheral detachment, instability, or a repairable tear. The present report also contributes sport-specific information regarding failed nonoperative management, postoperative rehabilitation, and return to athletic activity, details that have been inconsistently reported in previous pediatric cases. At 12 weeks after the procedure, the patient demonstrated full functional recovery and was cleared to return to sport, providing additional short-term evidence that isolated saucerization may produce favorable results when the peripheral rim remains stable. Although longer-term follow-up is needed, the absence of reported complications as of eight months postoperatively in this patient is consistent with the favorable outcome observed after isolated saucerization.

This case adds to the limited literature on discoid medial meniscus and supports arthroscopic saucerization as an effective treatment option in the absence of peripheral instability. Early recognition and appropriate surgical management are important for optimizing outcomes in this rare condition. Surgeons should maintain a high index of suspicion for discoid medial meniscus in young patients with persistent mechanical symptoms and atypical imaging findings.

Limitations

There are several limitations to this case report. Most notably, the single-patient design restricts the generalizability of our findings. Although case reports serve a valuable role in documenting rare clinical presentations and surgical decision-making, conclusions drawn from an individual case are not always applicable to the broader population of young athletes with discoid meniscus pathology.

The follow-up duration reported here is relatively short, limiting our ability to draw conclusions about the durability of the meniscus after return to sport or future complications resulting from the procedure. Discoid meniscus surgery in skeletally immature individuals carries unique long-term considerations, including the risk of recurrent tears, progressive chondral changes, and altered joint biomechanics, which can only be adequately assessed over years of follow-up rather than weeks or months. Although this patient returned to sport without complications at 12 weeks postoperatively and has had no reported complications as of eight months after the procedure, the lack of long-term follow-up or follow-up imaging substantially limits our ability to evaluate patient-reported outcomes or potential complications beyond this period.

Additionally, this report does not include standardized patient-reported outcome measures, such as the International Knee Documentation Committee Score, Knee Injury and Osteoarthritis Outcome Score, or Lysholm Knee Scoring Scale. Incorporation of these validated instruments would have provided a more objective and standardized assessment of functional recovery and quality of life and would have strengthened comparability with the existing literature.

## Conclusions

A discoid medial meniscus is a rare but clinically significant cause of mechanical knee symptoms in young athletes. Accurate diagnosis and meniscal-preserving surgical techniques are critical for optimizing outcomes. Arthroscopic saucerization can provide effective symptom relief, restore knee function, and facilitate return to sport while minimizing the long-term risk of degenerative changes.
